# Prevalence of pelvic floor disorder and associated factors among women in Arba Minch Health and Demographic Surveillance Site, Gamo Zone, Southern Ethiopia, 2021

**DOI:** 10.3389/fruro.2023.1196925

**Published:** 2023-09-11

**Authors:** Berhanu Negese Kebede, Desta Haftu Hayelom, Gebremaryam Temesgen Birgoda, Awol Arega Yimer, Bezawit Afework Mesfin, Mesfin Difer Tetema, Solomon Seyife Alemu, Kassaw Beyene Getahun

**Affiliations:** ^1^ Department of Midwifery, College of Medicine and Health Sciences, Arba Minch University, Arba Minch, Ethiopia; ^2^ School of Public Health, College of Medicine and Health Sciences, Arba Minch University, Arba Minch, Ethiopia; ^3^ Department of Midwifery, College of Medicine and Health Sciences, Wolkitie University, Wolkitie, Ethiopia; ^4^ Department of Midwifery, Madda Walabu University, Bale Robe, Ethiopia

**Keywords:** pelvic floor disorder, women, factors, Gamo Zone, southern Ethiopia

## Abstract

**Background:**

Globally, millions of women develop pelvic floor disorder. It imposes a considerable emotional, social, and financial burden on women’s lives. Despite this, in developing countries, nearly half of women with pelvic floor disorder do not seek any help due to feelings of isolation, depression, shame, and loss of control. Thus, the magnitude of the problem is largely unknown. The aim of this study was to assess the prevalence of pelvic floor disorder and associated factors among women at Arba Minch Health and Demographic surveillance site.

**Methods:**

A cross-sectional study with a simple random sampling technique was employed on a community basis. The data were entered into EpiData version 3.1 and then exported to Statistical Package for Social Sciences version 25 for data cleaning and analysis. Bivariate and multivariable analyses using binary logistic regressions were carried out to identify factors associated with pelvic floor disorder. The level of significance was declared at a *p*-value of < 0.05.

**Results:**

The prevalence of pelvic floor disorder was 31.4% (95% CI = 26.9% to 35.8%). Being grand multiparous (AOR = 3.919, 95% CI = 1.495–10.276), having a history of instrumental delivery (AOR = 3.042, 95% CI = 1.483 to 6.241), having a history of perianal tearing (AOR = 2.972, 95% CI = 1.491 to 5.927), and having a medical disease (AOR= 2.698, 95% CI = 1.526 to 4.770) were factors associated with pelvic floor disorder.

**Conclusions and recommendations:**

The prevalence of pelvic floor disorder was high in the study area. Parity, instrumental delivery, perianal tears, and medical problems were factors affecting the prevalence of pelvic floor disorder. There is a need for an improvement of policies and strategies focusing on prevention and treatment services to alleviate the problem.

## Introduction

Pelvic floor disorders (PFDs) are conditions that affect the proper function of a woman’s pelvic organs and can occur when the pelvic floor muscles are too weak, too tight, damaged, or overused (1).

PFDs comprise a collection of conditions, the most notable of which are urinary incontinence (UI), pelvic organ prolapse (POP), and fecal incontinence (FI) (2).

According to a joint report of an international urogynecological association (IUGA/International Continence Society), POP is defined as the descent of the anterior vaginal wall, posterior vaginal wall, uterus (cervix), or vaginal apex (3), and UI is any involuntary loss of urine (4). Fecal incontinence is defined as the involuntary loss of solid and liquid feces (3) and the inability to postpone an evacuation until it is socially acceptable (5).

Stress UI, urge incontinence, and mixed incontinence are all types of urinary incontinence (3, 4).

The prevalence of PFD among women in high-income countries is estimated to be between 25% and 43% (6, 7). The prevalence of POP alone was reported to be between 2.9% and 12.8% (6, 8, 9), with 3.5%–15% of women reporting fecal incontinence in high-income countries (6, 7).

According to a study conducted in developing countries, 20%, one-third, and 7% of parous women have POP, UI, and FI, respectively (10). In low-income countries, UI was reported at a rate of 25%–42% (11, 12), and the prevalence of POP ranged from 2% to 64.8% (13–16).

In Ethiopia, one-eighth to two-fifths of women have one form of PFD (17–19); however, the majority of women with PFDs do not have access to appropriate healthcare (10, 17). The findings of studies conducted in hospitals revealed a high prevalence of POP, with rates ranging from 13.3% to 40.7% (20–22); studies conducted at the community level reported a prevalence rate of 9.5% (17). This variation between the findings of community- and hospital-based studies also reflects a low level of healthcare seeking and the silent suffering of many women with a PFD (17).

The consequences of PFD issues can be disastrous (10). They can cause significant health problems (23), a negative impact on daily quality of life (11), physical activity restriction, emotional affection, and sleep deprivation in 90% of affected women, and role limitations in 70% (12). Women with a PFD, particularly POP, were fearful of disclosure, discrimination, and divorce as a result of community and family perceptions of shameful and strongly prohibited physical and social conditions (24).

In high-income countries, pregnancy and childbirth are well-known risk factors for PFDs (25–27). Furthermore, in developing countries, high parity, home delivery, age, and prolonged heavy lifting are among the factors that increase the risk of PFDs (16, 28).

Ethiopia is a low-income country with limited obstetric care (48% of births are institutionalized) and a high fertility rate (4.6 children per woman) (29). Women in the Arba Minch Health and Demographic Surveillance Site have limited access to obstetric care, putting them at a higher risk of obstetric-related pelvic floor injury, with 76.9% of all births taking place at home prior to 2015 (30). Despite this, many healthcare providers focus on the immediate complications of childbirth, while delayed maternal complications, which may take decades to develop as other lifestyle factors contribute to the development of these disorders, receive less attention. As a result, the purpose of this study was to determine the prevalence of pelvic floor disorder and its associated factors among women in the Arba Minch Health and Demographic Surveillance Site in Gamo Zone, southern Ethiopia.

## Materials and procedures

### Design and context of the study

From 1 to 30 May 2021, a community-based cross-sectional study was conducted at the Arba Minch Health and Demographic Surveillance Site in the Arba Minch Zuria and Gacho Baba districts, Gamo Zone, Southern Ethiopia. According to 2015 census projections, the districts had a total population of 164,529, of which 82,330 were women. The districts had 31 kebeles (the smallest administrative units) in total and were part of the Arba Minch Zuria Demographic and Health Development Program (AM-DHDP). Arba Minch Zuria Demographic and Health Development Program is owned by Arba Minch University, one of six public universities in Ethiopia that has a Health and Demographic Surveillance System (HDSS) (31). The surveillance site is made up of eight rural and one semi-urban kebele, selected from a total of 31 kebeles in the district. The Arba Minch Health and Demographic Surveillance Site contains 24,206 registered households.

### Populations

As a source population, all women in the Arba Minch Health and Demographic Surveillance Site were considered. Women registered in the Arba Minch HDSS database and living in the kebeles of the Arba Minch Health and Demographic Surveillance Site were the study populations.

### Criteria for eligibility

The study included women who were registered in the Arba Minch Health and Demographic Surveillance Site database. The study excluded pregnant women and mothers with a postpartum period of fewer than 6 weeks.

### Determining the sample size

The sample size was calculated using the single population proportion formula with the following assumptions: the proportion (p) of pelvic floor disorder in the Kersa district being 20.5% (17), 95% confidence level (, and the degree of precision being 4%. The minimum required sample size was calculated by substituting the above assumptions in the formula: 392. With a 10% response rate, the final minimum required sample size was 431.


$$\text{n}=\text{Z}\alpha /2\frac{\text{p}(1-\text{P})}{{\text{d}}^{2}}$$

Where

n = the required sample size;

z = the value of the standard normal curve score corresponding to the given confidence level, which is 1.96

p = proportion of pelvic floor disorder, which is 20.5%

d = the permissible margin of error, which is 4%

### Sampling method and procedure

The study took place in the Arba Minch Health and Demographic Surveillance Site’s nine kebeles. A simple random sampling technique was used to select the study sample, which was based on the ID numbers of registered households in each selected kebele. The total number of households in each kebele was determined using the Arba Minch Health and Demographic Surveillance Site database. To adjust the calculated sample size for each kebele, proportional sample allocations were made. Using the Arba Minch HDSS database as a sampling frame, the required number of target women was drawn at random from the sample frame. Finally, the ID numbers of selected households were extracted from the database and printed as hard copies for use in the field.

### Variables

#### Dependent variable

PFD.

#### Independent variables

Socio-demographic variables: age, residence, marital status, educational level, ethnicity, occupation, and income.

Obstetrics and gynecologic factors: gravidity, abortion, parity, age at first delivery, place of delivery, perianal tear and episiotomy, mode of delivery, and menopausal status.

Medical and personal factors: heavy lifting, lung disease, diabetes mellitus, urinary tract infection, kind of work or daily activity (working on a farm, carrying water, and preparing false bananas—”kocho”). Heavy physical work and heavy weight lifting increase intra-abdominal pressure, which is believed to play a role in the pathogenesis of PFD (specifically POP). Kocho, an Ethiopian flatbread or bread-like fermented food made from chopped and grated enset pulp, is a traditional and favorite food in the study area. Processing kocho/enset is one of the strenuous tasks women have to perform as one of their daily activities.

### Measurement

Women’s responses to questions about UI, POP, and anal incontinence were used to assess PFDs. The presence of the problem was defined by positive responses to at least one of the questions from each of the PFD categories. Women who reported at least one PFD symptom were classified as “having pelvic floor disorder” (17). A four-point Likert scale was used to assess the levels of distress caused by each PFD’s symptoms. If symptoms were present, the individual was asked, “How much are the symptoms bothering you?” And the response was graded on a scale of “not at all = 1” to “very seriously = 4”. The mean value of symptom distress was multiplied by 25 to obtain the score ranges of 0–100 for each domain of PFD. The severity of the symptoms among women with a PFD was determined based on distress score ranges from 1 to 100 and categorized by tertiles (into three parts) as “mild” if the total score was 1–33, “moderate” if the total score was 34–66, and “severe” if the score was 67–100 (17).

### Method, instrument, and procedure for data collection

Data were collected using semi-structured, interviewer-administered data collection tools.

Questions about pelvic floor symptoms were adapted from a previous study conducted in Ethiopia (11). The questions assessing other independent variables such as socio-demographic factors, obstetric and gynecologic history, and personal and medical history were adapted primarily from the Epidemiology of Prolapse and Incontinence Questionnaire (32). For data collection, nine midwives with a Bachelor of Science degree who were fluent in Amharic and Gamottho were hired. Three field supervisors with Masters’ degrees in clinical midwifery were also hired to monitor the process and the quality of the data collected.

### Data quality control

To ensure consistency, the questionnaire was prepared in English and translated into Amharic and back to English prior to data collection. A pretest was conducted on 22 women (5% of the sample) in the Mirab Abaya woreda. Data collectors received a 2-day training course supported by practical demonstrations on interview methods, with an emphasis on the introduction, communication to correctly assess outcomes, and respecting cultural norms in the community. During the data collection phase, the data collectors thoroughly checked the questionnaire’s completion. The principal investigator and supervisors provided daily intensive supervision throughout the data collection period.

### Processing, analysis, and interpretation of data

Data were entered into EpiData 3.1 before being exported to Statistical Package for Social Sciences (SPSS) version 25 for cleaning and further analysis.

To describe the characteristics of participants, descriptive statistical analyses such as simple frequencies, percentages, medians, and interquartile ranges were used. In the binary logistic regression model, the relationship between the outcome variable and several independent variables was first examined separately. In the second step, independent variables with a *p*-value of 0.25 in bivariate analysis were retained and entered into the multivariable logistic regression analysis together. The degree of association between the outcome and independent variables was determined using an OR with a 95% confidence interval and a *p*-value.

The statistical significance level was set at a *p*-value of < 0.05.

### Ethical consideration

The institutional review board of Arba Minch University’s College of Medicine and Health Science granted ethical clearance with the ethical clearance number IRB/1066/21. Before collecting data, each study subject provided written consent. Names and identification were not included in the written questionnaires to protect the confidentiality of the information. Each study subject was informed that their participation would be entirely voluntary during the data collection process. Women who were discovered to have pelvic floor symptoms during data collection but were not seeking treatment were counseled and referred to healthcare facilities.

## Results

### Socio-demographic characteristics

A total of 431 participants were approached and 427 eventually participated in the study, giving a response rate of 99.1%. The respondent’s median age was 29 years [interquartile range (IQR) = 24–45 years]. The majority of the respondents, 361 (84.5%), were in a marital union (married and living with their husband). The majority of respondents (402) (94.1%) were Gamo in ethnicity. Sixty-six percent of those polled were housewives, and 43.3% had no formal education (**Table 1**).

**Table 1:** Socio-demographic characteristics of the study participants of the study on prevalence of pelvic floor disorder and associated factors in Arba Minch HDSS, Southern Ethiopia, 2021.

Variable		Number (*N* = 427)	Percentage (%)
Age of respondents (years)	18–24	121	28.3
	25–34	128	30.0
	35–44	67	15.7
	45–54	36	8.4
	≥55	75	17.6
Marital status	In marital union	361	84.5
	Divorced	31	7.3
	Widowed	35	8.2
Residence	Rural	411	96.3
	Urban	16	3.7
Ethnicity	Gamo	402	94.1
	Wolaita	20	4.7
	Zeyise	5	1.2
Occupation	Housewife	283	66.3
	Laborer	53	12.4
	Employee	59	13.8
	Other (student, farmer, trader)	32	7.5
Educational status	No formal education	185	43.3
	Primary	112	26.2
	Secondary	99	23.2
	Diploma and above	31	7.3

### Reproductive history of respondents

Overall, 26.7% of the participants had experienced pregnancy five times or more. In addition, 32% of respondents had a history of abortion, with nearly half having had only one. Almost all study participants were parous, with 22.8% being grand multiparous.

Approximately half of those polled had experience with home delivery. Thirty-five percent of study participants had a history of episiotomy during labor, and 14.7% had a history of instrumental delivery. Regarding perianal tears, regardless of the place of delivery, 18.5% of respondents reported experiencing perianal tears during their delivery. Around 34.3% of respondents had a history of cesarean delivery, with roughly three-quarters having undergone only one cesarean delivery. Menopause had been experienced by 22% of those polled (**Table 2**).

**Table 2:** Reproductive characteristics of the study participants of the study on the prevalence of PFD and associated factors in Arba Minch HDSS, Southern Ethiopia, 2021.

Variables		Number (*N* = 427)	Percentage (%)
Number of pregnancies	1	136	31.9
	2–4	177	41.4
	≥5	114	26.7
History of abortion	Yes	139	32.6
	No	288	67.4
Frequency of abortion	1	69	49.6
	2	38	27.3
	≥3	32	23.1
Parity	Primiparous	162	38.0
	Multiparous	167	39.2
	Grand multiparous	97	22.8
Age at the first delivery (years)	15–18	144	33.8
	≥19	282	66.2
History of home delivery	Ever at home	207	48.6
	Never at home	219	51.4
Ever had a vaginal delivery	Yes	389	91.3
	No	37	8.7
Number of vaginal deliveries	1	167	42.9
	2–4	142	36.5
	≥5	80	20.6
History of episiotomy	Yes	136	35.0
	No	253	65.0
Instrumental delivery	Yes	57	14.7
	No	332	85.3
A perianal tear during delivery	Yes	72	18.5
	No	317	81.5
Cesarean delivery	Yes	146	34.3
	No	280	65.7
Frequency of caesarean section	1	106	72.6
	≥2	40	27.4
Menopause	Yes	93	21.8
	No	334	78.2

### Medical history and personal history

Forty-two (9.8%) respondents had a history of recurrent urinary tract infections, while 9.4% and 14.8% had diabetes mellitus and lung disease/asthma, respectively. The majority of respondents (90.4%) had been required to lift more than 9 pounds on a regular basis.

In terms of daily activity, 91.1% of respondents carried water for personal use on a daily basis.

Seventy percent of respondents carry water two to three times per day on average, while 65.3% and 37% were involved in farming and false banana preparation, respectively (**Table 3**).

**Table 3:** Medical and personal history of the study participants of the study on prevalence of PFD and associated factors in Arba Minch HDSS, Southern Ethiopia, 2021.

Variable			Number (*N* = 427)		Percentage (%)
History of UTI	Yes		42		9.8
	No		385		90.2
History of DM	Yes		40		9.4
	No		387		90.6
History of lung disease	Yes		59		14.8
	No		368		86.2
Do you now or have you in the past been required to lift more than 9 pounds regularly (excluding your children)?	Yes		386		90.4
	No		41		8.6
Concerning the kind of work, do you now or have you in the past been performing the following activities?,	Carrying water	Yes	389		91.1
		No	38		8.9
	Working on a farm	Yes	279		65.3
		No	148		34.7
	Preparing false bananas	Yes	158		37
		No	269		63

### Prevalence of pelvic floor disorder

**Figure 1:**
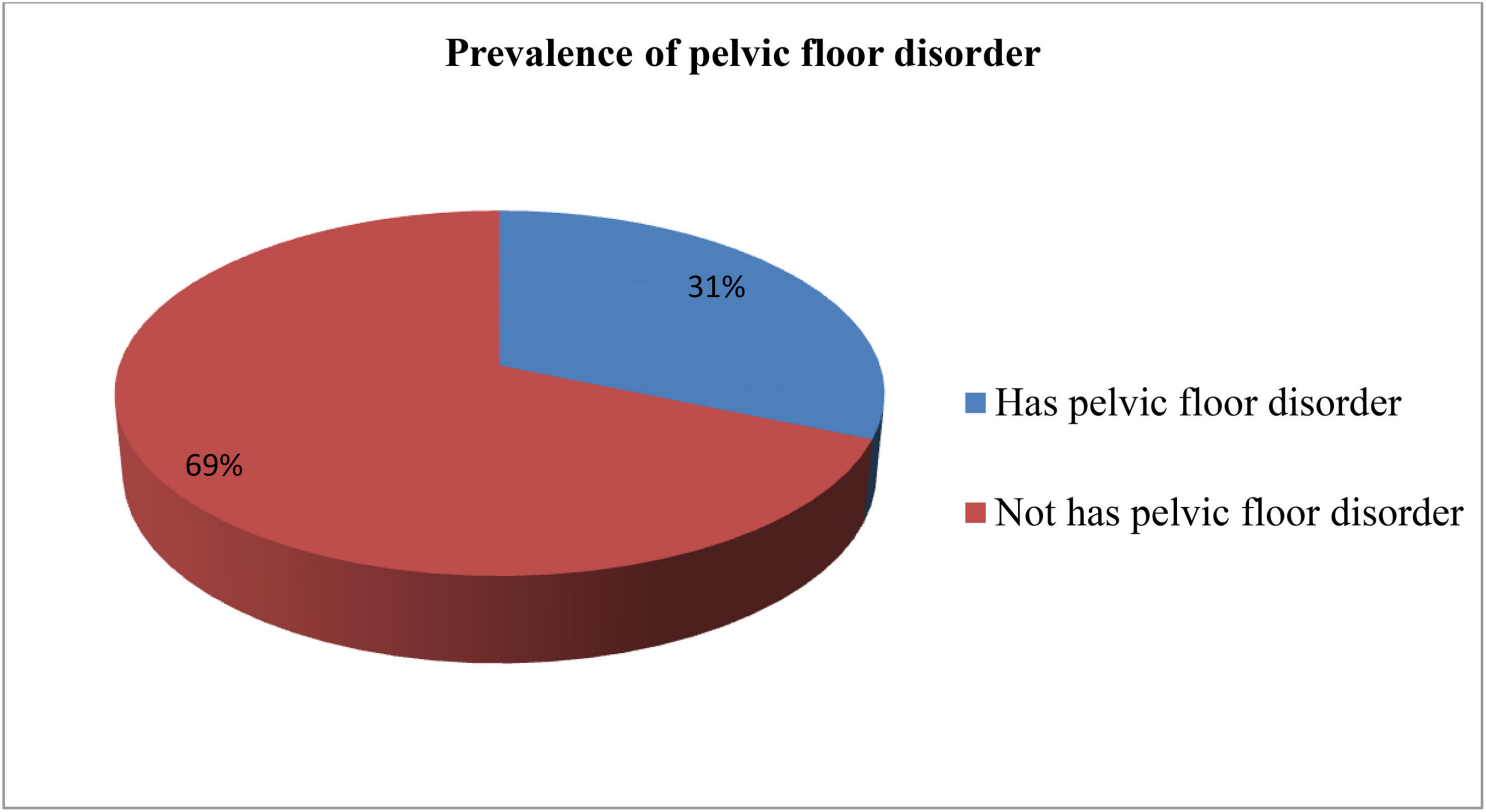
Overall prevalence of pelvic floor disorder among study participants in Arba Minch Health and demographic surveillance site, 2021. Regarding co-occurrence of pelvic floor disorder 19.7%.

Overall, 134 (31.4%, 95% CI = 26.9% to 35.8%) of the women reported at least one form of PFD (**Figure 1**). Incontinence was reported by 122 (28.6, 95% CI = 24.3% to 32.8%) of respondents. The prevalence of each PFD (and corresponding 95% CI) was 19.2% (15.5% to 22.9%) for urge incontinence, 11.2% (8.2% to 14.2%) for stress UI, 8.2% (5.6% to 10.8%) for mixed UI, 12.2% (9.1% to 15.3%) for anal incontinence, and 9.8% (6.9% to 12.6%) for POP.

Among those who had a PFD, 57.5% reported being seriously worried about their symptoms, whereas 39.6% of women were moderately bothered about their symptoms.

**Figure 2:**
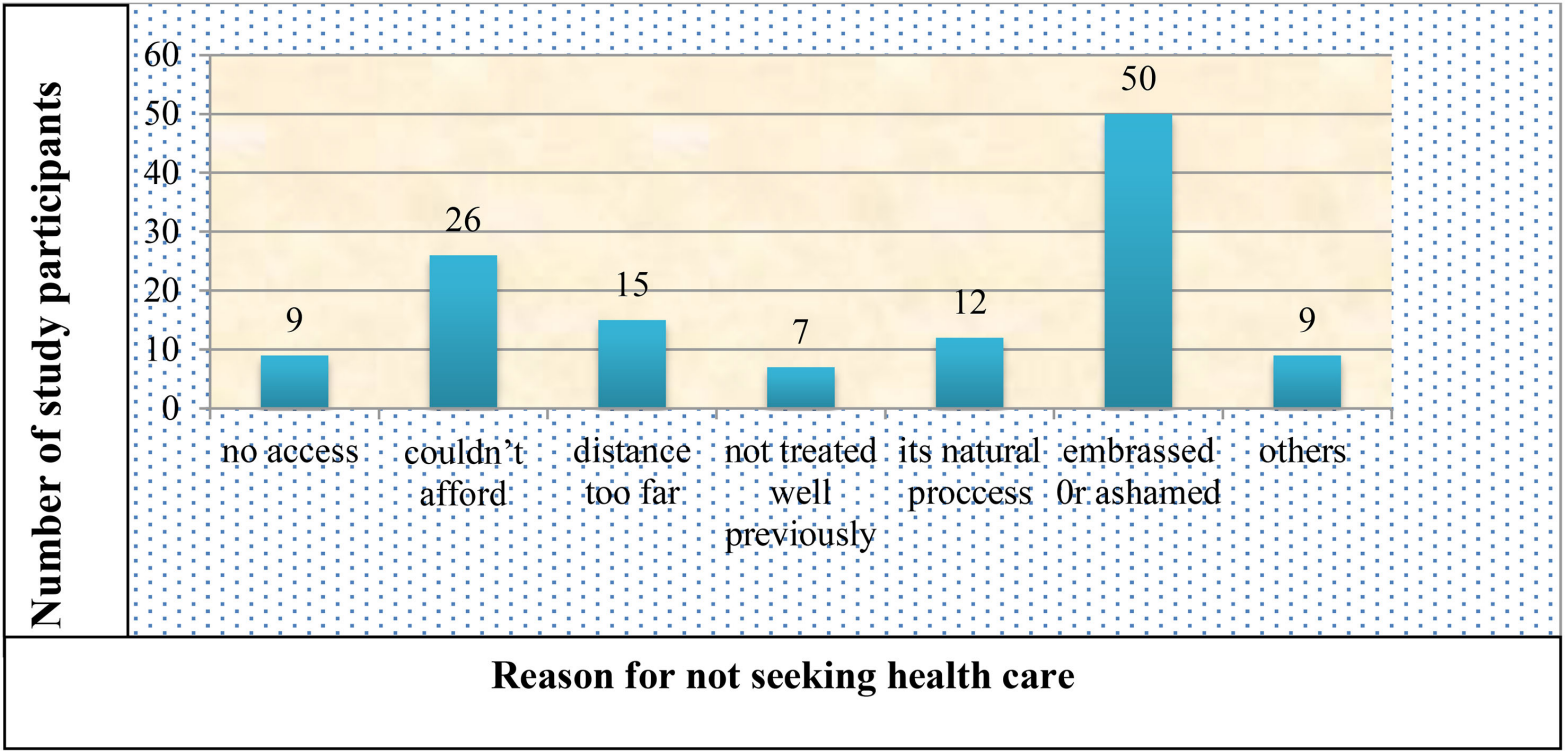
Reason for not seeking health care for pelvic floor disorder among the study participants in Arba Minch HDSS, 2021. Others include transportation problems, not ever seen by the health care provider and no female health care providers.

Only 36% of those who reported having a PFD sought medical attention for their symptoms. The most frequently reported reasons for not seeking healthcare were feeling embarrassed/ashamed about the symptoms, being unable to afford it, believing it was a natural part of the aging process, and being too far away (**Figure 2**).

### Factors associated with pelvic floor disorder

For the bivariable logistic regression, all variables were computed separately, and variables with a *p*-value of 0.25 were retrieved and entered into a multivariable logistic regression analysis. The respondents’ age and the number of vaginal delivery variables were excluded because they were highly correlated with the number of delivery variables in the linear logistic regression collinearity diagnostics. Four of the ten variables included in the multivariable logistic regression analysis showed a significant association.

According to the findings, being grand multiparous (AOR = 3.919, 95% CI = 1.495 to 10.276), having a history of instrumental delivery (AOR = 3.042, 95% CI = 1.483 to 6.241), having a history of perianal tearing (AOR=2.972, 95% CI = 1.491 to 5.927), and having a medical disease (AOR=2.698, 95% CI = 1.526 to 4.770) were factors associated with PFD (**Table 4**).

**Table 4:** Bivariate and multivariable logistic regression analyses as a result of factors associated with PFDs among study participants in Arba Minch HDSS, Ethiopia, 2021.

Variables		Has PFD	Has no PFD	COR (95%CI)	AOR (95%CI)	*p*-value
Marital status	In marital union	100	261	1.00	1.00	
	Divorced	10	21	1.243 (0.565 to 2.732)	0.488 (0.178 to 1.336)	0.163
	Widowed	24	11	^a^5.965 (2.690 to 12.055)	2.033 (0.797 to 5.183)	0.137
Occupation	Housewife	98	185	1.00	1.00	
	Laborer	17	36	0.891 (0.476 to 1.668)	0.895 (0.391 to 2.044)	0.792
	Employee	12	47	^a^0.482 (0.244 to 0.951)	0.528 (0.221 to 1.263)	0.151
	*Others	7	25	0.529 (0.221 to 1.226)	1.133 (0.401 to 3.199)	0.813
Parity	Primiparous	21	141	1.00	1.00	
	Multiparous	48	119	^a^2.708 (1.535 to 4.779)	1.913 (.902 to 4.058)	0.091
	Grand multiparous	65	32	^a^13.638 (7.308 to 24.453)	^b^3.919 (1.495 to 10.276)	0.005
Ever delivered at home	Yes	98	109	^a^4.57 (2.915 to7.165)	1.811 (0.905 to 3.623)	0.093
	No	36	183	1.00	1.00	
Had history of episiotomy	Yes	39	97	1.00	1.00	
	No	87	166	^a^1.304 (0.828 to 2.051)	1.308 (0.663 to 2.581)	0.439
Ever had instrumental delivery	Yes	37	20	^a^5.051 (2.784 to 9.164)	^b^3.042 (1.483 to 6.241)	0.002
	No	89	243		1.00	
Ever had perianal tear	Yes	48	24	^a^6.128 (3.526 to 10.65)	^b^2.972 (1.491 to 5.927)	0.002
	No	78	239	1.00	1.00	
Medical problems	Yes	62	45	^a^4.746 (2.981 to 7555)	^b^2.698 (1.526 to 4.770)	0.001
	No	72	248	1.00	1.00	
History of heavy lifting	No	6	35	1.00	1.00	
	Yes	128	258	^a^2.894 (1.187 to 7.058)	2.302 (0.711 to 7.458)	0.164
Preparing false banana	No	71	198	1.00	1.00	
	Yes	63	95	^a^1.849 (1.217 to 2.81)	1.306 (0.759 to 2.247)	0.335

a Indicates variables that were candidates in the bivariate analysis.

b Indicates variables that are significant in the multivariable analysis.

A COR or AOR of 1.00 indicates the reference category in the model.

## Discussion

The prevalence of PFD in this study was 31.4%. This result was higher than a study done in the United States of America (6) and India (14) which reported a prevalence of 25% and 21% respectively. Childbirth is reported to be a well-known risk factor for PFDs (25–27). Ethiopia is among the countries with limited obstetric care (institutional delivery is at 48%) (33) and a high fertility rate (4.6 children per woman) (29). Therefore, the observed difference in the prevalence of pelvic floor disorders could also be partly attributed to the difference in access to obstetric care services and the difference in fertility rates between the countries.

The prevalence of PFD in this study was also higher than the reported 20.5% and 11.9% overall prevalence of PFD in the Kersa (17) and Dabat (18) districts, respectively. The possible reason could be socio-demographic differences among study participants, as the proportion of laborers accounted for 12.6% of this study population but only 2.5% in the Dabat district study population. Prolonged heavy lifting, believed to play a role in the pathogenesis of POP by increasing intra-abdominal pressure, is identified as a risk factor for PFD in developing countries (16, 28, 34). Thus, there could also be other contributing factors for an increase in the prevalence of PFD; more than 90% of study participants were engaged in heavy lifting activities on a regular basis, and more than one-third were involved in preparing false bananas (kocho) in the study area.

The result of this study was lower than that of a study conducted in Iran, which revealed a 42% prevalence of one form of PFD (13). A reported low prevalence could be due to differences in the study population, study setting, and data collection tools. The former was carried out at a hospital and incorporated physical examinations into data collection.

According to the findings of our study, the prevalence of mixed UI was 8.2%. This was in line with the findings of a study conducted in eastern part of Ethiopia, which reported a prevalence of 7.7% for mixed UI (17). However, it was lower than the findings of a study carried out in South-Central Ethiopia, which reported a prevalence of 14% for mixed UI (19). This discrepancy could be owing to the differences in the study population, as the latter included pregnant mothers. This finding in our study was higher than the finding of a study carried out in Norway, which reported a prevalence of 5.9% for mixed UI (35). This difference might be owing to the fact that the Norwegian study was conducted among women younger than 65 years and excluded those who had had more than four vaginal deliveries.

In this study, parity, history of institutional delivery, history of instrumental delivery, history of perianal tearing, previous history of UI during pregnancy, and having medical problems were factors associated with the prevalence of PFD.

The odds of having a PFD were 3.919 times higher among grand multiparous women than primiparous women. This was supported by findings of a study in Germany and Denmark (36), India (12), and Tanzania (16). This could be due to the weakening and damage of the pelvic floor by the excessive strain and stretching caused by repeated births.

Instrumental delivery and perianal lacerations increased the risk of PFDs. Women with at least one forceps delivery and perianal lacerations in two or more deliveries were reported to have increased risks of a PFD (37). This is in accordance with the finding of this study, which revealed that the odds of a PFD were 3.042 times higher among women who had a history of forceps or vacuum deliveries than among those who did not (37). Similarly, the odds of having a PFD were 2.972 times higher among women who had at least one perianal tear than among those who did not have a perianal tear. These birth-related risks could be due to compression, stretching, or the tearing of nerve, muscle, and connective tissue related to vaginal delivery, as intact neuromuscular function and pelvic organ support are both critical to the normal function of pelvic viscera (38).

The prevalence of PFDs was found to be lower in the healthy population group than in those with medical comorbidities (39). Chronic obstructive pulmonary disease (COPD) (36), bronchial asthma (40), and diabetes mellitus have all been linked to an increase in the prevalence of PFDs (41, 42). This is consistent with the study’s findings, which showed that the odds of having a PFD are 2.698 times higher in people who have a history of lung disease, diabetes mellitus, or urinary tract infections than in people who have never had any of these medical problems. Health education on prevention strategies, behavioral change communication to improve community health-seeking behavior, and family planning promotion could all be beneficial.

## Conclusion

Women in the study area had a high prevalence of PFDs. Factors associated with PFDs included parity, instrumental delivery, perianal tearing, and medical problems. To alleviate the suffering of women with a PFD, policies and strategies focusing on prevention and treatment services must be improved.

## Data availability statement

The raw data supporting the conclusions of this article will be made available by the authors, without undue reservation.

## Ethics statement

The studies involving humans were approved by the Arba Minch University College of Medicine and Health Science Institutional Review Board. The studies were conducted in accordance with the local legislation and institutional requirements. The participants provided their written informed consent to participate in this study.

## Author contributions

BK, DH, and GB conceived the study and undertook the statistical analysis. AY, SA, and MT supervised the study and statistical analysis. BM and KG contributed to the writing of the manuscript. All authors contributed to the article and approved the submitted version.
