# Supplementary material for: Prevalence of pelvic floor disorder and associated factors among women in Arba Minch Health and Demographic Surveillance Site, Gamo Zone, Southern Ethiopia, 2021
Source: Front Urol. 2023 Sep 11;3:1196925. doi: 10.3389/fruro.2023.1196925 (PMC12327235; doi:10.3389/fruro.2023.1196925)
Supplement: Supplementary file 1 [file DataSheet_1.pdf]

Survey tool

Part I. questions assessing socio-economic characteristics of respondents in Arbaminch HDSS, Gamo zone, Southern Ethiopia; 2021

s.no	Question	Response	Option	Skip
**101**	Age in completed years	In years ………	[____]	
**102**	Marital status	In union ……...<br>Divorced …….<br>Widowed ……	1<br>2<br>3	
**103**	Where is your residence	Rural …………<br>Urban …………	1<br>2	
**104**	Your ethnic group?	Gamo……….<br>Wolaita …….<br>Zeysie………<br>Other specify……	1<br>2<br>3<br>4	
**105**	What is your occupation?	Housewife……<br>Farmer<br>Laborer....<br>Private Employee….<br>Governmental employee…..<br>Other specify ……..	1<br>2<br>3<br>4<br>5<br>6	
**106**	What is the higher education you have attained?	Has no formal education<br>Primary<br>Secondary<br>Diploma and above<br>Other specify….	1<br>2<br>3<br>4<br>5	
**107**	Household monthly income	In ETB_________		

Part II. Questions assessing obstetrics and gynecological factors among women women in Arbaminch HDSS, Gamo zone, Southern Ethiopia; 2021

**s. no**	**Question**	**Response**	**Option**	**Skip**
**201**	Age at first marriage	______years		
**202**	Have you ever been pregnant?	Yes<br>No	1<br>2	222
**203**	How many times have you been pregnant?	In number	[__|_]	
**204**	Have you ever had an abortion?	Yes<br>No	1<br>2	206
**205**	How many abortions did you have?	In number___		
**206**	Have you ever delivered a baby?	Yes<br>No	1<br>2	221
**207**	How many babies you deliver?	In number	[__|_]	
**208**	What was your age at your first delivery?	Year____	[__|_]	
**209**	Have you ever deliver a baby at home?	Yes<br>No	1<br>2	
**210**	Have you ever delivered a baby vaginally?	Yes<br>No	1<br>2	215
**211**	How many babies have you delivered vaginally?	In number	[__|__]	
**212**	Did you ever have a forceps or vacuum placed on the head of the baby to help deliver your baby vaginally?	Yes<br>No<br>I don't know	1<br>2<br>99	

**213**	Did you ever have an episiotomy or intentional cut on the vagina at the time of delivery?	Yes<br>No<br>I don't know	1<br>2<br>99	
**214**	Did you have a ''tear'' in the anal sphincter or muscle?	Yes<br>No<br>I don't know	1<br>2<br>99	
**215**	Did you ever have delivery by Caesarean section?	Yes<br>No	1<br>2	217
**216**	How many Cesarean sections have you had?	In number	[__|__]	
**217**	Have you experienced menopause?	Yes<br>No<br>I don't know	1<br>2<br>99	301
**218**	Approximately how old were you when you began menopause?	In year<br>I don't know	[__|__]<br>99	

Part III questions assessing about general health of participants in Arba Minch HDSS, Gamo zone, Southern Ethiopia; 2021

**s.no**	**Question**	**Response**	**Option**	**Skip**
301	Have you ever been diagnosed with any of the medical conditions listed below? (Please circle yes, no, or I don't know for each.)			
302	Urinary tract or bladder infections (more than 3 in a year)	Yes<br>No<br>I don't know	1<br>2<br>99	
**303**	Diabetes	Yes<br>No	1<br>2	

		I don’t know	99	
**304**	Lung disease/Asthma	Yes<br>No<br>I don’t know	1<br>2<br>99	
**305**	Do you now or have you in the past been required to lift more than 9 pounds regularly (excluding your children)?	Yes<br>No<br>I don’t know	1<br>2<br>99	308
**306**	Approximately how many years did you repeatedly lift heavy things regularly?	In year	[__|__]	
**307**	Concerning the kind of work, do you now or have you in the past been performing the following activities? ,			
**308**	Carrying water	Yes<br>No	1<br>2	
**309**	How often did you engage in the day on average?	Once a day<br>Twice a day<br>Three<br>Specify	1<br>2<br>3<br>4	
**310**	Working on the farm	Yes<br>No	1<br>2	
**311**	Preparing false banana or ‘kocho’	Yes<br>No	1<br>2	

Part IV question assessing symptoms of pelvic floor disorder among respondents in Arba Minch HDSS, Gamo zone, Southern Ethiopia; 2021

**s. no**	**Question**	**Response**	**Option**	**Skip**
**401**	Do you rush to urinate so that you will	Yes	1	403

	not have leakage of urine?	No	2	
**402**	How much are you bothered by the need to rush to urinate?	Not at all<br>Mildly<br>Moderately<br>Greatly	1<br>2<br>3<br>4	
**403**	Do you awaken during your normal sleeping hours to urinate?	Yes<br>No	1<br>2	405
**404**	How much are you bothered by the need to get up at night to empty your bladder?	Not at all<br>Mildly<br>Moderately<br>Greatly	1<br>2<br>3<br>4	
**405**	Do you experience frequent urination?	Yes<br>No	1<br>2	407
**406**	How much are you bothered by frequent urination?	Not at all<br>Mildly<br>Moderately<br>Greatly	1<br>2<br>3<br>4	
**407**	Do you experience urine leakage related to a feeling of urgency?	Yes<br>No	1<br>2	409
**408**	How much are you bothered by urine leakage related to a feeling of urgency?	Not at all<br>Mildly<br>Moderately<br>Greatly	1<br>2<br>3<br>4	
**409**	Do you experience urine leakage related to activity, coughing, or sneezing?	Yes<br>No	1<br>2	411
**410**	How much are you bothered by urine leakage related to activity, coughing, or sneezing?	Not at all<br>Mildly<br>Moderately	1<br>2<br>3	

		Greatly	4	
**411**	Do you experience small amounts of urine leakage (drops)?	Yes<br><br>No	1<br><br>2	
**412**	How much are you bothered by small amounts of urine leakage (drops)?	Not at all<br><br>Mildly<br><br>Moderately<br><br>Greatly	1<br><br>2<br><br>3<br><br>4	
**413**	If you have experience urine leakage, urgency, or difficulty of voiding have you ever sought any health care services?	Yes<br><br>No	1<br><br>2	415
**414**	Have you had any surgeries or procedures to correct urine leakage?	Yes<br><br>No	1<br><br>2	
**415**	Do you have a sensation that there is a bulge in your vagina or that something is falling out from your vagina?	Yes<br><br>No	1<br><br>2	417
**416**	How much are you bothered by this sensation or bulge?	Not at all<br><br>Mildly<br><br>Moderately<br><br>Greatly	1<br><br>2<br><br>3<br><br>4	
**417**	If you have prolapse, have you ever sought any health care service for it?	Yes<br><br>No	1<br><br>2	419
**418**	Have you had any surgery to correct pelvic prolapse?	Yes<br><br>No	1<br><br>2	
**419**	Do you lose gas from your rectum that is beyond your control?	Yes<br><br>No	1<br><br>2	421
**420**	How much are you bothered by losing gas from your rectum?	Not at all<br><br>Mildly<br><br>Moderately<br><br>Greatly	1<br><br>2<br><br>3<br><br>4	

**421**	Do you lose stool beyond your control if your stool is loose or liquid?	Yes<br>No	1<br>2	423
**422**	How much are you bothered by losing loose or liquid stool?	Not at all<br>Mildly<br>Moderately<br>Greatly	1<br>2<br>3<br>4	
**423**	Do you lose well-formed stool beyond your control?	Yes<br>No	1<br>2	426
**424**	How much are you bothered by the loss of well-formed stool?	Not at all<br>Mildly<br>Moderately<br>Greatly	1<br>2<br>3<br>4	
**425**	Have you ever sought health care to correct any symptoms of anal incontinence?	Yes<br>No	1<br>2	
**426**	Why didn't you seek health care for these symptoms? (more than one answer is possible)	No access to health care workers.<br>Couldn't afford<br>Distance too far<br>Transportation problem<br>The opposition of care /Partner prohibition<br>Scare health care workers<br>Have not ever seen by health care workers<br>Not treated well previously	1<br>2<br>3<br>4<br>5<br>6<br>7<br>8	

		Embarrassed or ashamed	9
		No female health care Workers	10
		Assuming it as a natural part of the aging process	11

Thank you for your participation!!!
